# Ketamine-Induced Oscillations in the Motor Circuit of the Rat Basal Ganglia

**DOI:** 10.1371/journal.pone.0021814

**Published:** 2011-07-29

**Authors:** María Jesús Nicolás, Jon López-Azcárate, Miguel Valencia, Manuel Alegre, Marta Pérez-Alcázar, Jorge Iriarte, Julio Artieda

**Affiliations:** 1 Neurophysiology Laboratory, Neurosciences Area, CIMA, University of Navarra, Pamplona, Spain; 2 Clinical Neurophysiology Section, Clínica Universidad de Navarra, Pamplona, Spain; Chiba University Center for Forensic Mental Health, Japan

## Abstract

Oscillatory activity can be widely recorded in the cortex and basal ganglia. This activity may play a role not only in the physiology of movement, perception and cognition, but also in the pathophysiology of psychiatric and neurological diseases like schizophrenia or Parkinson's disease. Ketamine administration has been shown to cause an increase in gamma activity in cortical and subcortical structures, and an increase in 150 Hz oscillations in the nucleus accumbens in healthy rats, together with hyperlocomotion.

We recorded local field potentials from motor cortex, caudate-putamen (CPU), substantia nigra pars reticulata (SNr) and subthalamic nucleus (STN) in 20 awake rats before and after the administration of ketamine at three different subanesthetic doses (10, 25 and 50 mg/Kg), and saline as control condition. Motor behavior was semiautomatically quantified by custom-made software specifically developed for this setting.

Ketamine induced coherent oscillations in low gamma (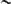
50 Hz), high gamma (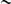
80 Hz) and high frequency (HFO, 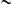
150 Hz) bands, with different behavior in the four structures studied. While oscillatory activity at these three peaks was widespread across all structures, interactions showed a different pattern for each frequency band. Imaginary coherence at 150 Hz was maximum between motor cortex and the different basal ganglia nuclei, while low gamma coherence connected motor cortex with CPU and high gamma coherence was more constrained to the basal ganglia nuclei. Power at three bands correlated with the motor activity of the animal, but only coherence values in the HFO and high gamma range correlated with movement. Interactions in the low gamma band did not show a direct relationship to movement.

These results suggest that the motor effects of ketamine administration may be primarily mediated by the induction of coherent widespread high-frequency activity in the motor circuit of the basal ganglia, together with a frequency-specific pattern of connectivity among the structures analyzed.

## Introduction

The synchronization of neuronal activity may be mechanistically important for information processing across different levels of the sensory and motor systems [1], [2]. Synchronized activity within a large population can be detected as oscillatory activity in the EEG/MEG or in local field potentials (LFP) recordings.

Changes in oscillatory activity might be related to the pathophysiology of neurological and psychiatric diseases. Altered oscillatory activity (suggesting the presence of abnormalities in neuronal synchronization) has been reported in schizophrenia, particularly around the gamma range [3]. Abnormal synchronization has also been found in other neurological diseases, such as Parkinson's disease, where an exaggerated synchronization exists in the beta frequency band between the motor cortex and different basal ganglia nuclei, with changes to the gamma range after dopaminergic intake [4], [5], [6],[7].

Ketamine is a pharmacological antagonist of NMDA glutamate receptors. It can induce hallucinations and paranoia comparable with the positive symptoms of schizophrenia, and can also cause behaviors similar to the negative symptoms of schizophrenia, like social withdrawal, poverty of speech and blunted affect [8]. Ketamine administration has been proposed as a potential animal model of schizophrenia in rodents. In rats, subanaesthetic doses have been described as producing hyperlocomotion, altered social interaction, sterotypies and impaired cognitive function. Some of these behaviors are similar to those observed in schizophrenic patients, suggesting that NMDA receptor hypofunction might contribute to some of the symptoms of schizophrenia [9]. In particular, it has been proposed that the hyper-locomotion observed might be the consequence of cognitive and perceptive disturbances mimicking the cognitive dysfunction observed in schizophrenic patients.

Ketamine administration in rats and mice generates changes in brain oscillatory activity at different frequencies: low doses (2.5–10 mg/Kg) of the drug induce an increase in gamma activity (30–80 Hz) in cortical and subcortical areas in otherwise healthy rats [10]. The increase in gamma power correlates with the hyperactivity observed [11], and may be accompanied by a theta power decrease [12], [13]. The presence of gamma alterations in both schizophrenic patients and animals treated with ketamine might support the validity of ketamine administration as a pathophysiological model of the disease. Hunt et al also reported the presence of high frequency oscillations in the Nucleus Accumbens (a ventral component of the striatum) around 140–160 Hz, which increased notably after a subanesthesic (10–50 mg/Kg) dose of ketamine [14], also accompanying hyperkinetic behavior.

The neocortex and basal ganglia, operating via parallel cortico-striatal-thalamocortical pathways, are involved in motor control and cognition [15], [16], [17]. Oscillatory activity is a key feature of these circuits, both in normal physiology and in the pathophysiology of several disorders. Cortico-striatal and cortico-subthalamic input into the basal ganglia circuitry is mediated by glutamate [18]. The subthalamic nucleus, a key structure in the basal ganglia circuitry commonly used as therapeutic target in Parkinson's disease, is also glutamatergic [19], [20], [21]. Some of the motor or behavioral effects observed after the induction of changes in glutamatergic transmission (by ketamine or other modulators of glutamate action) might be therefore due to the alterations in the function of cortical-basal ganglia circuits.

The aim of our study was to understand how ketamine modifies the dynamics of the motor circuit of the basal ganglia and its potential relationship with the observed hypermotoricity. We simultaneously recorded the oscillatory activity from the motor cortex and several structures of the motor circuit of the basal ganglia (caudade putamen, subthalamic nucleus and substantia nigra pars reticulata) from free moving healthy rats. Then, we analyzed the oscillatory activity induced by several doses of ketamine, and studied how the interrelations established between the basal ganglia nuclei and the cortex are modulated.

## Results

### Power spectra

**Figure 1:**
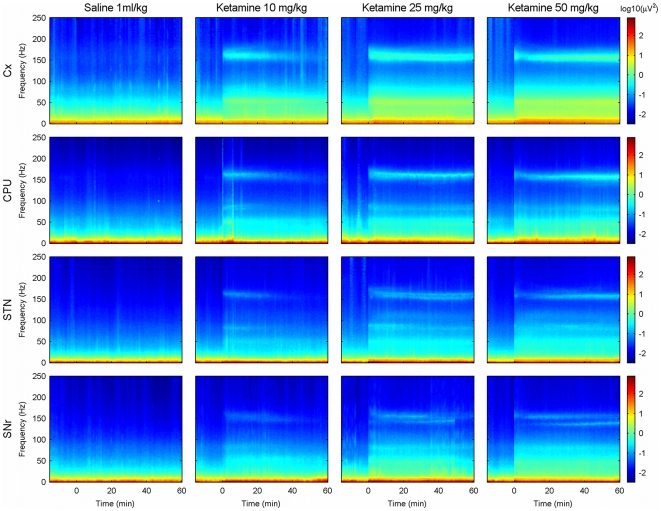
Time-frequency evolution of the ketamine-induced spectral changes. Time-frequency representation of the oscillatory activity recorded under the effect of saline injection and different doses of ketamine (columns) in the motor cortex, caudate-putamen, subthalamic nucleus and substantia nigra pars (rows). A short-time Fourier transform in the range of 0 to 250 Hz was computed for each animal, nuclei and condition. Then, a grand-average of all animals was obtained (see Methods). Saline/ketamine injection took place at time 0. Saline injection did not induce representative changes in any time or frequency. Post-ketamine administration periods were characterized by the irruption of oscillatory activities mainly focused in the low-gamma (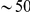 Hz), high-gamma (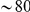 Hz) and high frequency ranges (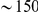 Hz).

Figure 1 shows the time-frequency representation of the spectral content along time for the four different brain locations and ketamine doses.

**Figure 2:**
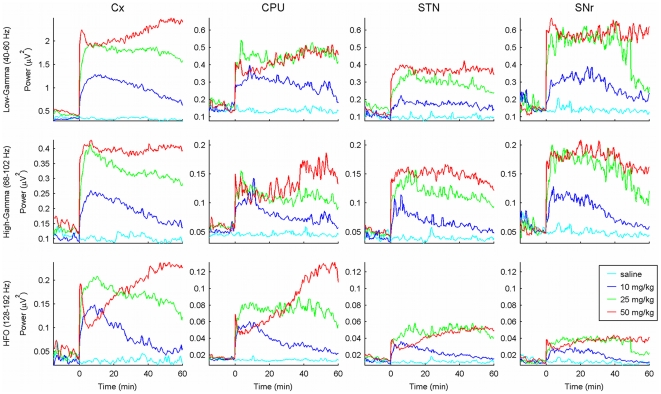
Time-varying spectral evolution of the 50, 80 and 150 frequency bands. The lines represent the grand-average of the mean power in the three frequency bands studied (50, 80 and 150 Hz) in the four nuclei recorded. Minute 0 is the time of injection. While saline injection did not affect the power in the analyzed bands, ketamine administration showed time-varying dose-dependent changes in the three bands.

Before ketamine injection, and after saline injection, the power spectra were similar in the four structures in relative terms, although motor cortex presented the highest absolute values in power. Qualitatively, oscillatory activity in the four structures were very similar under the basal and control (saline injection) conditions (Figure 1, first column; Figure 2, cyan traces). They were characterized by the classical reduction in power at increasing frequencies. Additionally, some animals (9/18 rats in motor cortex, 6/17 animals in CPU, 4/13 in STN and 5/11 in SNr) presented a small peak in the high frequency range, around 150–170 Hz.

After ketamine administration, the spectral content of the signals changed dramatically. Recordings were characterized by the presence of low-amplitude/high-frequency oscillations intermingled with high- amplitude/slow frequency activities. These changes were clearly reflected in the spectrogram, where it was possible to detect the appearance of different bands of oscillatory activity (see Figure 1 second, third and fourth columns). More specifically, ketamine induced a power increase in the low gamma (mean: 51,74 Hz, SD: 4,90 Hz), high gamma (mean: 84,56 Hz, SD: 3,55 Hz) and high frequency (mean: 160,55 Hz, SD: 8,07 Hz) bands.

Different doses of ketamine elicited distinct patterns of evolution in the time-course of the power recorded for the three frequency bands (Figure 2). After the 10 mg/Kg ketamine dose, all structures showed a fast increase in power in the relevant bands. The maximum values were achieved in the first ten minutes and then all structures presented a gradual power decrease, almost reaching baseline values at the end of the recording time (Figure 2, dark blue traces). Under the 25 mg/Kg ketamine dose all structures experienced a very fast increase in the power of the three frequencies followed by a plateau that lasted until 40–50 minutes after the injection. Thereafter, the power of the three bands declined gradually. This power increase was, in all cases, higher than the increase produced by the 10 mg/Kg dose. (Figure 2, green trace). Finally, the response to the highest ketamine dose (50 mg/Kg) was characterized by a biphasic pattern with a very fast increase, followed by a sharp decrease and a gradual increase maintained till the end of the recording (Figure 2, red trace).

In the low gamma range (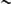 50 Hz), saline injection did not change the power in any of the four structures (cyan trace in Figure 2, first row). However, the administration of different doses of ketamine produced a rapid increase in power. Among the four structures, the motor cortex presented the largest changes, while the STN changed the least (Figure 2, first row, first and third column). An evident dose dependence was observed: higher doses of ketamine elicited higher power increases. In addition, while the increase caused by the doses of 10 and 25 mg/Kg of ketamine decayed along the recording time, the power values for the highest dose (50 mg/Kg) remained *in crescendo* (motor cortex and CPU) or constant (STN and SNr) until the end of the recording. The repeated-measures two-way ANOVA detected significant effects for the dose and time factors, and significant interaction between them (see Table 1). Post hoc analysis (Bonferroni-corrected 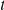-Student test) showed significant differences along the whole recording for 25 and 50 mg/Kg of ketamine comparing with saline condition, while the dose of 10 mg/Kg lost the significance around minute 40.

**Table 1:** Repeated measures two-way ANOVA analysis for the power.

	factors	Low Gamma	High Gamma	HFO
		(40–60 Hz)	(68–102 Hz)	(128–192 Hz)
	dose	F 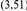 = 109.119**	F 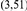 = 97.342**	F 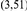 = 71.205**
Cx	time	F 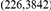 = 42.442**	F 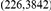 = 24.744**	F 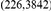 = 20.833**
	interaction	F 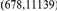 = 15.619**	F 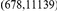 = 9.935**	F 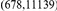 = 8.827**
	dose	F 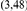 = 38.389**	F 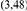 = 27.314**	F 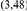 = 41.768**
CPU	time	F 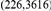 = 16.512**	F 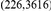 = 17.346**	F 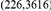 = 21.146**
	interaction	F 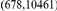 = 5.047**	F 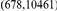 = 4.586**	F 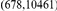 = 10.928**
	dose	F 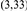 = 13.441**	F 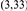 = 15.453**	F 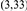 = 28.012**
STN	time	F 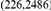 = 14.860**	F 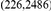 = 17.870**	F 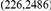 = 7.903**
	interaction	F 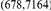 = 5.603**	F 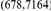 = 4.991**	F 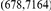 = 5.413**
	dose	F 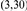 = 18.698**	F 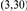 = 18.968**	F 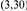 = 19.448**
SNr	time	F 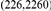 = 26.214**	F 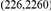 = 20.232**	F 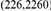 = 11.669**
	interaction	F 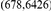 = 8.685**	F 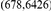 = 7.208**	F 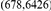 = 5.534**

**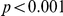.

*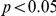.

The activity in the high gamma range (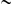 80 Hz) also experienced a fast increase after ketamine injection. In this frequency range, power values were lower than in the low gamma band (Figure 2, second row, note that the scales are different). Motor cortex showed the maximum power values, followed by substantia nigra, subthalamic nucleus and finally caudate-putamen. The temporal evolution for the three doses was very similar to that recorded for the low gamma band. The repeated-measures ANOVA also detected very significant effects in dose, time and an interaction between both factors (See Table 1). As it happened in the low gamma band, post hoc tests showed significant differences for the highest doses (25 and 50 mg/Kg) along recording time. For the dose of 10 mg/Kg, power values also decayed to baseline levels at the end of the recordings, losing the statistical differences compared with the saline condition around minute 20–30.

However, the most significant differences after drug administration were found in the high frequency band. High frequency oscillations (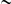 150 Hz) were detected in all the motor cortex recordings (18/18), in 15/17 CPU, 10/11 STN and 9/11 SNr. Although all ketamine doses (10, 25 and 50 mg/Kg) induced an increase in the power of the high frequency oscillations, important differences in the temporal evolution of the peak were found (see Figure 1 and Figure 2, third row). The lowest dose (10 mg/Kg) caused a transient increase that lasted for about 30 minutes. The 25 mg/Kg dose induced a larger increase in high-frequency power which decayed slowly, maintaining values well over baseline at the end of the recording. The highest dose (50 mg/Kg) generated a sharp increase in high-frequency oscillations (similar to the effect of the 25 mg/Kg dose), followed by a transient decrease (partial) and a slower increase sustained until the end of the recording. Power values after 30 minutes were higher than after any of the other doses. The statistical analysis showed significant differences for both factors, dose and time, and interaction between them. (See Table 1). Post hoc analysis showed significant differences after the three doses of ketamine. In case of 25 and 50 mg/Kg these differences were maintained along the recording hour but 10 mg/Kg of ketamine lost its signification around minute 20–30 depending on the nucleus.

### Imaginary coherence

**Figure 3:**
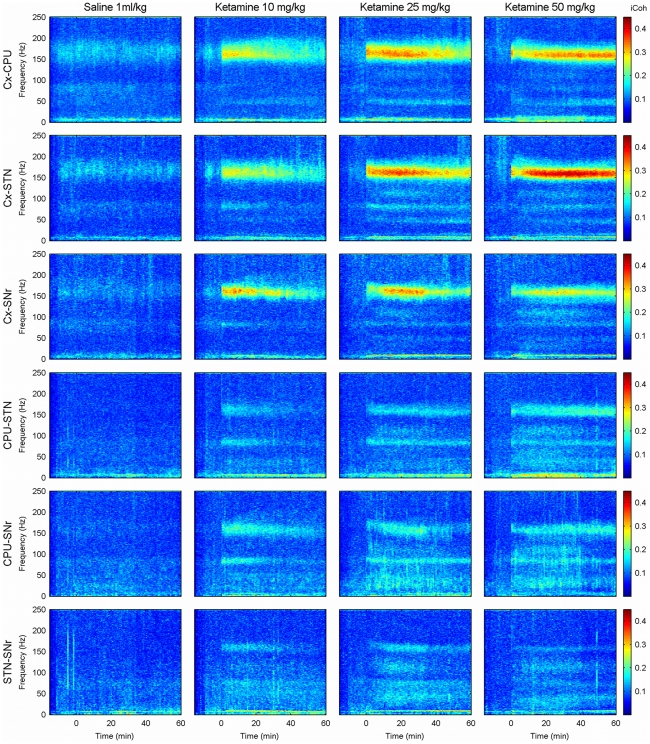
Ketamine-induced changes in inter-nuclear interactions. Grand-average of the time frequency evolution of the imaginary coherence under the effect of saline injection and three different doses of ketamine (columns) in the six possible links: Cx-CPU, Cx-STN, Cx-SNr, CPU-STN, CPU-SNr and STN-SNr (rows). Saline/ketamine was injected at time 0. No changes in imaginary coherence were observed after saline injection. However, ketamine administration induced marked changes in the imaginary coherence in the low gamma (50 Hz), high gamma (80 Hz) and high frequency (150 Hz) bands. Links between the cortex and the basal ganglia showed a very marked increase in coherence in the HFO band. In contrast, links between basal ganglia nuclei were more coherent in the gamma range.

Imaginary coherence was studied on all six possible pairs of structures recorded, namely, the interactions between Cx-CPU, Cx-STN, Cx-SNr, CPU-STN, CPU-SNr, and STN-SNr were analyzed (Figure 3).

**Figure 4:**
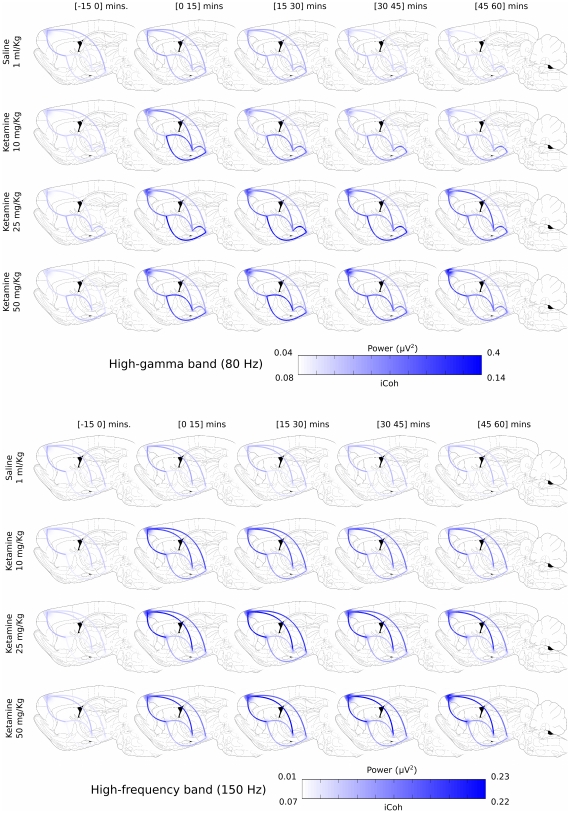
Changes in the dynamics of the oscillatory activity for the 80 and 150 Hz bands. Power is represented as a big dot over each nucleus with different color intensity according to its values. The lines between nuclei display the imaginary coherence between each pair of structures. As in power values, color intensity indicates different levels of coherence. Interactions between cortex and basal ganglia nuclei showed a very marked increase in coherence in the HFO band after ketamine. In contrast, the connections between the basal ganglia nuclei became more coherent in the high gamma range.

While ketamine administration induced an increase in the power spectrum of all structures at the three different frequency bands (50, 80 and 150 Hz), the increase of interactions observed showed a different topology for each band. Figure 4 shows, for the two frequency bands with the most significant changes (80 and 150 Hz), a composite representation where the time evolution of power and imaginary coherence appear simultaneously in the different structures studied.

Saline administration did not increase the coherence at any nucleus/frequency band (Figure 3, leftmost column).

In the low gamma band (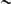 50 Hz), ketamine induced small increases in the imaginary coherence values, observed in all pairs. The largest effect was detected under the 50 mg/Kg of ketamine (Figure 3, rightmost column). The repeated-measures two-way ANOVA showed a significant effect of the dose factor only in Cx-STN, Cx-SNr and CPU-STN pairs, where dose of 50 mg/Kg induced the most significant differences in coherence values with respect to those observed in saline condition. Anyway significant dose
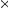
time interaction effects were present in all pairs (see Table 2).

**Table 2:** Repeated measures two-way ANOVA analysis for the imaginary coherence.

	factors	Low Gamma	High Gamma	HFO
		(40–60 Hz)	(68–102 Hz)	(128–192 Hz)
	dose	F 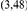 = 0.73	F 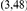 = 0.451	F 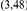 = 15.169**
Cx-CPU	time	F 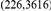 = 3.242**	F 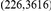 = 1.031	F 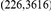 = 23.245**
	interaction	F 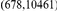 = 1.367**	F 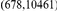 = 1.105	F 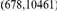 = 6.537**
	dose	F 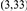 = 5.508*	F 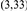 = 2.134	F 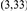 = 11.330**
Cx- STN	time	F 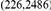 = 3.516**	F 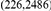 = 2.168**	F 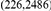 = 15.052**
	interaction	F 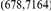 = 1.443**	F 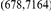 = 1.308**	F 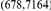 = 3.746**
	dose	F 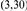 = 4.089*	F 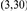 = 0.780	F 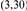 = 8.206**
Cx-SNr	time	F 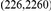 = 1.309*	F 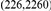 = 0.785	F 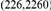 = 10.839**
	interaction	F 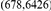 = 1.123*	F 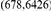 = 1.664**	F 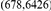 = 2.959**
	dose	F 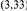 = 3.189*	F 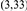 = 7.408**	F 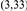 = 10.154**
CPU-STN	time	F 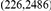 = 1.681**	F 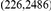 = 4.038**	F 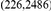 = 7.350**
	interaction	F 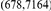 = 1.203**	F 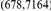 = 1.713**	F 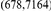 = 2.861**
	dose	F 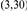 = 1.414	F 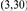 = 1.665	F 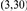 = 5.847*
CPU-SNr	time	F 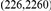 = 2.465**	F 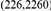 = 4.152**	F 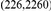 = 7.883**
	interaction	F 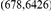 = 0.986	F 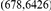 = 1.224**	F 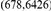 = 2.817**
	dose	F 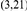 = 0.689	F 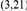 = 0.478	F 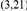 = 4.542*
STN-SNr	time	F 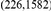 = 3.095**	F 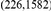 = 3.004**	F 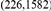 = 4.207**
	interaction	F 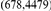 = 1.334**	F 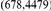 = 1.305**	F 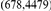 = 1.746**

**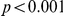.

*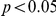.

Ketamine administration caused a rapid increase in the imaginary coherence in the high gamma band (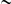 80 Hz) in the six pairs of interactions (Figure 3), but the increase was more evident in the pairs connecting basal ganglia nuclei than in those involving the motor cortex (Figure 4, top). This differential effect was best observed with the lowest dose of ketamine (second row in Figure 4). The effect of the 10 mg/Kg dose was sustained for 30–35 minutes, while the other two doses maintained high levels of coherence till the end of the recording (See Figure 3 and Figure 4, top). The statistical analysis (see Table 2) showed a significant effect of dose only in the CPU-STN pair, where dose of 50 mg/Kg of ketamine produced a significant increase of the coherence values comparing to saline condition. Significant dose 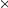 time interactions were found in Cx-STN, Cx-SNr, CPU-STN, CPU-SNr and STN-SNr pairs.

Significant increases in coherence in the high frequency band (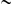 150 Hz) were observed in all pairs of nuclei (Table 2). Among all frequencies, this was the band where the highest values of imaginary coherence were detected. Roughly, the temporal evolution of the coherence values was similar to that of the power spectrum peaks described in the previous section. Nevertheless, some exceptions were observed: the lowest dose of ketamine (10 mg/Kg) induced significant changes (respect to saline condition) that decayed gradually in all nuclei until losing their significance around 30–40 minutes after the injection (Figure 3, second column and Figure 4, bottom). After the 25 mg/Kg dose, mean coherence values for the high frequency oscillations were higher than those for the 10 mg/Kg dose, with less marked decay. Moreover, some pairs showed a plateau until minute 30–40 followed by gradual decrease until the end of the recordings (Figure 3, third column). For the 50 mg/Kg dose, coherence values did not decrease, but remained at high values for the whole recording (Figure 3, fourth column). At this frequency, for the three doses, the highest imaginary coherence values were observed in the pairs involving the motor cortex (Cx-CPU, Cx-STN and Cx-SNr), while basal ganglia nuclei pairs showed lower coherence values (Figure 4, bottom).

### Locomotor activity

**Figure 5:**
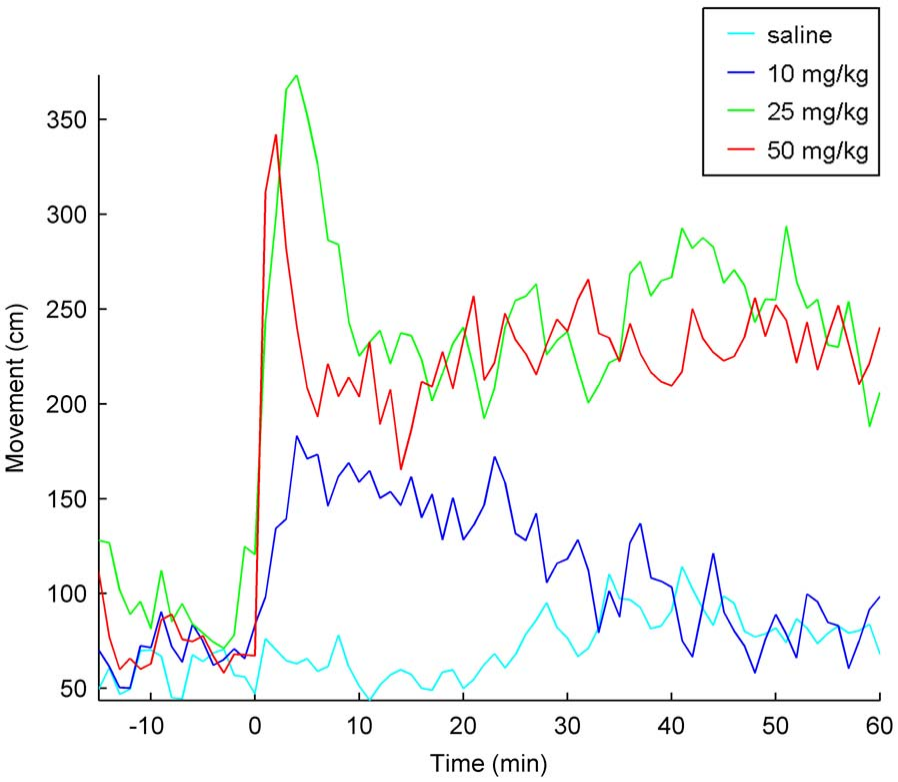
Ketamine-induced dose-dependent increase in locomotor activity. Saline/ketamine was administered at time 0 after a 15 minutes habituation period. Ketamine injection produced dose-dependently increases in the locomotor activity.

During the pre-injection period and after saline solution administration, animal movement was sporadic. Ketamine injection was accompanied by a marked increase in locomotor activity, with hyperactivity, circling and head-weaving. The temporal evolution of the motor activity was different after each dose. The onset of the hyperactivity was immediate for the highest doses, 25 and 50 mg/Kg, while it took two or three minutes for the 10 mg/Kg dose (Figure 5).

After 10 mg/Kg injection (figure 5, dark blue), locomotor activity reached its maximum level of hyperactivity around minute 3, when it started to decay smoothly till the end of the recording. After 40 minutes, motor activity was similar to that observed after saline administration.

Higher doses of ketamine (25 and 50 mg/Kg) induced higher degrees of hyperactivity that lasted till the end of the recording period. Motor activity had a similar temporal evolution after both doses: an initial increase, followed by a transient decay (with a shape similar to that observed in the decrease of the high-frequency power). Then locomotor activity recovered around minute 20 and was sustained at high values up to the end of the recording.

The statistical comparison, repeated measures two ways ANOVA, showed significant differences in locomotor activity due to drug effect (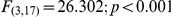), time effect (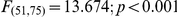) and interaction (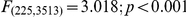). Post hoc analysis revealed that these differences in case of 10 mg/Kg of ketamine were lost during minute 25, while the highest doses of drug (25 and 50 mg/Kg) maintained the statistical differences till the end of the recording.

### Correlation between movement and power/coherence values

**Figure 6:**
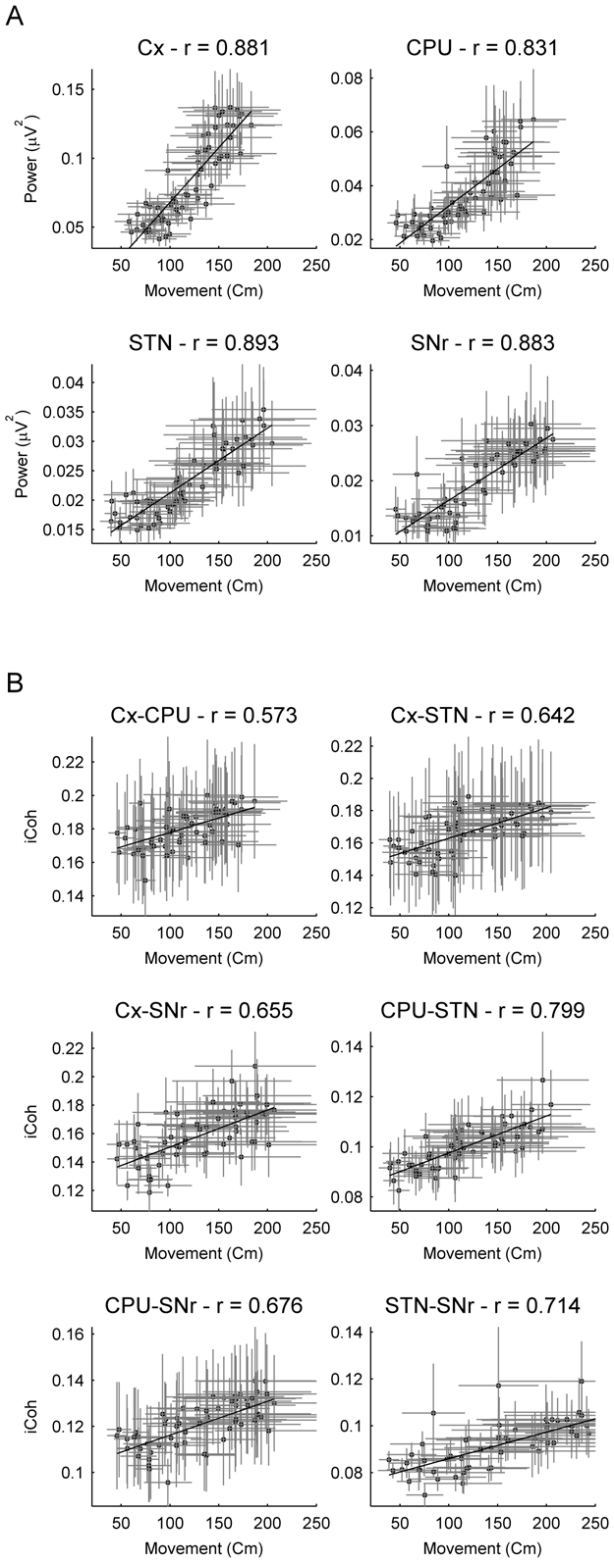
Linear correlation between ketamine-induced changes in power/iCoh and locomotor activity. (A) Pearson's correlation between the power of the high frequency (150 Hz) band and the locomotor activity under the 10 mg/kg ketamine dose showed a very significant correlation (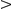
0.8) in the four structures. (B) Linear correlation between the imaginary coherence of the high frequency (150 Hz) band and the locomotor activity under the effect of 10 mg/kg ketamine. The six links showed a highly significant positive correlation.

Locomotor activity after ketamine administration showed a positive correlation with the increase of power (Table 3) and imaginary coherence (Table 4) values in the three frequency bands analyzed (low gamma, high gamma and high-frequency) in most nuclei. Nevertheless, correlation estimates in the gamma band were lower than those in the high frequency band for both, power and coherence. The highest correlations were observed for the lowest ketamine dose where the Pearson coefficient achieved values close to 0.9 (Table 3 and Figure 6, top).

**Table 3:** Linear correlations between movement and Power values for the 10 mg/Kg ketamine dose.

	Low Gamma	High Gamma	HFO
	(40–60 Hz)	(68–102 Hz)	(128–192 Hz)
Cx	r = 0.842**	r = 0.865**	r = 0.881**
CPU	r = 0.594**	r = 0.615**	r = 0.831**
STN	r = 0.541**	r = 0.778**	r = 0.893**
SNr	r = 0.767**	r = 0.896**	r = 0.883**

**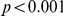.

*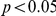.

**Table 4:** Linear correlations between movement and iCoh values for the 10 mg/Kg ketamine dose.

	Low Gamma	High Gamma	HFO
	(40–60 Hz)	(68–102 Hz)	(128–192 Hz)
Cx-CPU	r = −0.343*	r = −0.11	r = 0.572**
Cx-STN	r = 0.168	r = 0.603**	r = 0.642**
Cx-SNr	r = 0.014	r = 0.456**	r = 0.655**
CPU-STN	r = 0.352*	r = 0.765**	r = 0.799**
CPU-SNr	r = −0.03	r = 0.627**	r = 0.676**
STN-SNr	r = 0.1378	r = 0.094	r = 0.714**

**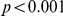.

*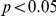.

Although lower, correlations between power and movement were also highly significant for the gamma range. Correlations in the low gamma range were sightly lower than those of the high gamma, with Cx and SNr achieving the highest values in both frequency ranges.

Regarding the imaginary coherence estimates, correlations reached very significant levels for all the pairs in the HFO range. In the high gamma range, interactions between pairs Cx-STN, Cx- SNr, CPU-STN and CPU-SNr presented a very good correlation with movement, while on the other hand, there was a certain degree of independence between interaction strength values and movement in the low gamma range.

## Discussion

Subanesthesic doses of ketamine induced coherent oscillations in the gamma (30–80 Hz) and high frequency (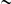
150 Hz) ranges. The use of spectral techniques together with the analysis of imaginary coherence allowed us to detect differences in the topographic organization of the activities as well as different roles related to the observed hypermotoricity.

### Gamma activity

Enlarged gamma oscillations (30–80 Hz) in cortical and subcortical structures after giving NMDA antagonists had already been reported in a series of studies with rats by Hakami et al. [11]. These authors described an increase in gamma activity after low doses of ketamine in several cortical and subcortical structures, which correlated with hypermotoricity. Our approach allowed us to identify two different frequency peaks within the gamma band, one centered around 50 and the other in the 80 Hz range, extending these findings to the whole motor circuit. As the two gamma frequency ranges showed different correlations and topographical characteristics, they will be discussed separately.

#### Low gamma band (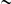 50 Hz)

In our rats, ketamine administration induced an increase in the amplitude of low gamma oscillations (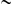
50 Hz) for all doses; imaginary coherence estimates (maximum between the cortex and the striatum) did not present a direct (at least linear) relation with movement.

Low gamma oscillations have been related to normal sensory and cognitive processing [22], [23]. Several studies have decribed differences in low gamma oscillations in schizophrenic patients when compared to healthy controls [3], [24], [25], [26],[27]. In fact, impairment in the ability of distributed networks to establish precise synchronization of neuronal assemblies oscillating at high frequencies when performing cognitive processes might be a core factor in the pathophysiology of schizophrenia [28], [24], [29], [25]. Altogether, these findings suggest that the increase of low gamma activity after ketamine administration might be related to cognitive dysfunction more than to the hypermotoricity experimented by the animals.

#### High gamma band (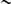 80 Hz)

We also found a ketamine-induced peak of coherent activity in the high gamma range (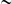
80 Hz). Inter-structural interactions mediated by high gamma oscillations were more constrained to the basal ganglia nuclei and had an evident relationship with the motor behavior of the animals.

Local field potential studies carried out in human patients have shown widespread activity in the high gamma range in the motor circuit of the basal ganglia, including the thalamus [4], [6], [30]. This activity increases in power and coherence during voluntary movements, suggesting a role for these frequencies in motor control [31]. Cortico-subcortical coherence in this range seems to be led by subcortical structures, as opposed to lower frequencies [32]. All these evidences suggest a role for high gamma activity in the hypermotoricity observed in our animals, mediated through subcortical structures.

Human studies mostly carried out in patients with Parkinson's disease, show that high gamma activity in the basal ganglia is greatly enhanced by dopaminergic stimulation [4], [33]. Ketamine, besides being a NMDA antagonist, might have some D2 dopaminergic agonist activity. This could indicate that the enhancement of high gamma oscillations in the basal ganglia after ketamine administration is mediated by D2 dopaminergic activation. Indeed, the administration of D2 agonists induces an increase in the high gamma oscillations in the basal ganglia of healthy rats similar to that observed in our study [34], [35]. However, Hakami et al. show that MK801, a “pure” NMDA antagonist, also causes an increase in gamma activity similar to the increase observed after ketamine [11]. In any case, optogenetic studies suggest that the mechanism of gamma oscillations is an emergent property from coupled networks composed of excitatory and inhibitory cells [36], so imbalances in the excitation/inhibition equilibrium (as those induced by NMDA activation) could give rise to pathologic activities.

### High frequency activity (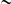 150 Hz)

The frequency range that showed the maximal relative increase after ketamine administration in all the structures studied was around 150 Hz. The largest amplitudes were detected over the motor cortex while striatum, subthalamic nucleus and substantia nigra pars reticulata (all of them part of the motor circuit of the basal ganglia) presented lower amplitudes at these frequencies. Previous studies have already described the effects of ketamine over high-frequency oscillations in subcortical structures, mainly in the Nucleus Accumbens [37]. Our results present temporal profiles that are very similar to those published by Hunt et al [14].

We also detected the presence of high frequency activites in some of the rats in basal condition, but after ketamine injection, the amplitude of these oscillations was notably increased. This finding suggests on the one hand, a physiological origin for these activities and on the other hand, that ketamine increases them, suggesting a potential relationship between high-frequency oscillations and the hypermotricity observed. The changes in locomotor activity observed in our study had a temporal dynamics similar to the high frequency power or coherence plots in all studied doses. The maximum correlation between locomotor activity and 150 Hz power suggests that the hyperactivity observed might be mediated by the presence of increased high-frequency activity and interrelations between the motor cortex and the basal ganglia circuits.

High-frequency oscillations (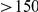 Hz) are observed during normal activity of different brain regions, including cortical evoked potentials [38], [39] or cerebellar oscillations [40], [41]. High frequency oscillations (around 300 Hz) can be recorded in the human subthalamic nucleus in patients with Parkinson's disease [42], [43]. This activity is present at rest, but it is better modulated when patients make a voluntary movement after L-DOPA administration. It has also been detected in the subthalamic nucleus of non-parkinsonian patients for frequencies greater than 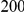 Hz [44], or in subdural electrodes located over healthy tissue from patients undergoing neurosurgical treatment for epilepsy [45]. All these findings will point to a physiological role for the high frequency oscillations in corticostriatal circuits.

The description of highly synchronized activity between the rat cortex and the basal ganglia in this frequency range (150 Hz) is novel. The choice of the imaginary coherence as a tool to unveil the presence of functional connectivity ensures that the interactions here described are mostly mediated by motor cortex and that they are not due to other sources of activity or to spurious volume conduction effects. Although the origin of the oscillatory activity within the basal ganglia circuits is not well known, it is very likely that it will share some of the mechanisms proposed above, which are probably the consequence of complex interactions between different structures. The differences observed between the motor cortex and the other three subcortical structures suggest that cortical circuits could be playing an important role in the generation and transfer of this kind of activities.Excitatory projections from the cortex to the subthalamic nucleus and striatum are glutamatergic, and NMDA receptors modulate the efficiency of glutamatergic excitation, so it seems reasonable to think that NMDA antagonists (like ketamine) will be able to affect the dynamics of the system and therefore the emergent oscillatory activities.

Our findings can be summarized by concluding that ketamine induces at least three specific changes in the activity of both the motor cortex and in the motor portion of the basal ganglia nuclei. First, our results define the appearance of two peaks in the gamma range after low-dose ketamine administration. One is around 50 Hz, predominates in motor cortex and CPU, and correlates only sightly with hypermotoricity. Previous findings in EEG studies in control subjects and schizophrenic patients suggest that this peak may potentially be related to cognitive disturbance. The other component is around 80 Hz and predominates in the basal ganglia with a good correlation with movement; its similarity to the activities described in Parkinsons disease patients after dopaminergic stimulation suggests that it may be due to the activation of D2 receptors by ketamine. Finally, our results show that the induction of high-frequency activity by ketamine (150 Hz) becomes widespread and affects the whole motor circuit of the basal ganglia with a high level of correlation with motor behavior. This fact would point to a cardinal role for the motor cortex in the increase of motoricity observed after ketamine administration.

## Materials and Methods

The effect of different doses of ketamine on the oscillatory activity of the motor cortex and three nuclei from the basal ganglia (STN, SNr, Caudate-putamen) was studied in 20 adult male Wistar rats (250–300 gr).

Two animals were excluded from further analysis because of the death of the animal before the recording protocol was completed (one case) and overall bad signal quality (another case).

### Ethics Statement

The whole protocol was approved by the institutional animal ethics committee; Comité de Ética para la Experimentación Animal, Universidad de Navarra, approval ID 088-06.

### Surgery for electrode implantation

Rats were anesthetized with ketamine (75 mg/Kg i.p.) and xylazine (11 mg/Kg i.p.) and positioned in a stereotaxic frame. Blunt ear bars were used to avoid any damage in the tympanic membrane of the animals.

A single midline incision was made over the scalp and six holes were drilled thought the scull for the implantation of the electrodes. Two different types of electrodes were used in order to record local field potentials. Concentric electrodes with two contacts (0.25 mm, Model SNE-100, Kopf Instruments, Tujunga, California, USA) were stereotactically placed in the subthalamic nucleus, CPU and SNr. Due to the asymmetry in the contact areas of the electrode, the selected set up can be considered an asymmetric bipolar recording with an active contact located on the tip (inner contact) locally referred to the outer (larger) contact. Cortical local field potentials were recorded by means of stainless steel screws (1.6 mm diameter, Plastics One, Roanoke, VA, USA, ref. E363) placed in the skull. Active electrode was placed in the primary motor cortex referred to an electrode placed in the auditory cortex. An additional screw placed in the frontal region was used as ground electrode. The wires of the electrodes were connected to a custom-made small ten-channel socket that was firmly held with dental cement (Faciden, Olot, Spain) to the rat skull. Only the terminal male pins of the socket were uncovered by the skin.

The stereotactical coordinates for the electrode placement were selected according to the atlas by Paxinos and Watson (1998): [anterior(AP): 2.70 mm and lateral(L): 3.20 mm] for the motor cortex, [AP: −4.8 mm and L: 7.4 mm] for the auditory cortex (reference for motor cortex recording), [AP: 0.20 mm and L: 3 mm, V: −6 mm] for caudate-putamen, [AP:−3.80 mm, L: 2.5 mm, V: −7.8 mm] for STN and finally [AP: −5.80 mm, L: 2 mm, V: −8 mm] for SNr. The location of the electrodes was confirmed post mortem by a histological analysis with thionine staining.

The animals were then placed in separate cages for four to five days recovery with food and water prior to the pharmacological experiments. Antibiotic (enrofloxacin, Alsir 10%, Esteve, Spain) was administrated orally for one week to avoid infections. Postoperative analgesia was also administered (Ketoprophen, 2 mg/Kg sc, Ketofen 1%, Lab, Spain).

### ECoG, LFP and locomotion in freely moving rats

The recording procedure started from four to five days after electrode implantation.

Animals were recorded inside a custom-made Faraday cage shielded from external electrical fields and were connected to the recording equipment by two cables which hanged from the top of the cage (Ref. 363–363 50 cm 6TCS, spring, Plastics One, Roanoke, VA, USA). A multi-channel rotatory commutator (SL12C/SB, Plastics One, Roanoke, VA, USA) was used to allow the animals free movement inside the cage.

Three different sub-anaesthetic doses of ketamine (Ketolar 50 mg/ml, Pfizer, Madrid, Spain) administered by intraperitoneal injection, were used: 10, 25 and 50 mg/Kg. At these concentrations, animals were awake during the whole recording. Saline solution (0.9%, B Braun, Barcelona, Spain) administered intraperitoneally was used as control.

Each recording began 45 minutes after the connection of the cables in order to let the animals become used to the Faraday cage. Animals were initially recorded for 20 minutes before the ketamine (or saline) injection to obtain baseline oscillatory activity. Animals were then disconnected for the injection procedure, reconnected and recorded for further 60 minutes. It took between one and two minutes to re-connect the animals after the injection.

All recordings were carried out in the same order for all the animals, and they extended for three days. The first day, saline and 10 mg/Kg ketamine recordings were carried out, with a three-hour interval between the end of the saline recording and the beginning of the ketamine recording. On the second day the animals were recorded before and after 25 mg/Kg ketamine injection, and on the third day the response to the 50 mg/Kg ketamine dose was recorded.

Animal movement was tracked by a webcam located at the top of the cage throughout all the recording procedure. To do this, we measured the position of the animal's head in a semi-automatic way for every single frame of the video recording. This allowed us to measure the total displacement of the head in the time periods of interest (every single minute in this case) by means of a custom-made software running under Matlab (Mathworks, Natick, MA, USA) in 1 minute-length blocks.

### Signal conditioning

The signal was filtered 0.3–1000 Hz and amplified x20000 using Grass P511 amplifiers (Grass, W Worwick, RI, USA). A CED power 1401 A/D converter (CED, Cambridge, UK) was used to acquire the signal with a sampling frequency of 2500 Hz.

Misplaced electrodes (detected in posterior histological analysis) and recording channels with suboptimal signal quality were also excluded from further analysis, giving a total of 18 motor cortex, 17 CPU, 13 STN and 11 SNr studied.

### Analysis of oscillatory changes

Changes in spectral content were assessed by means of the Welch's periodogram. This method for estimating power spectra is carried out by dividing a signal into successive segments that are weighted by an appropriate window function to avoid spectral leakage. Next, the periodogram of each segment is calculated by computing the discrete Fourier transform, and then computing the squared magnitude of the result. The individual periodograms are then time-averaged, which reduces the variance of the individual power measurements. To obtain a complete description of the temporal evolution of the spectral content, here we computed the Welch's periodogram in sliding windows (blocks) of 60 seconds with 20 seconds of overlap.

Briefly, each block 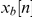 of 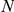 samples length is segmented into 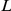 non-overlapping segments,
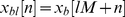
(1)where 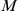 is the segment length (here 1 second). Each segment is then weighted with a Hanning window 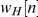, and its Fourier transform computed
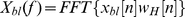
(2)where 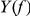 is the Fast Fourier Transform (FFT) implementation of the Fourier Transform for the signal 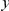. Finally, the block-spectrogram is estimated as:
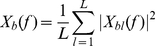
(3)


Merging each of these 1-minute block spectrograms (using a Hanning window of 1 second length) results in a 2D representation that determines the spectral content of local sections of the signal as it changes over time.

### Analysis of connectivity changes

The functional relationships between the motor cortex and the basal ganglia nuclei were estimated by means of the *imaginary coherence* (iCoh). This measure has been shown to be insensitive to false connectivity arising from volume conduction, identifying only true brain interactions at different frequency bands, reflecting the oscillatory properties of the brain electrical activity [46].

Probably the simplest and most popular measure of ‘interaction’ at a specific frequency is *coherence*, a generalization of correlation to the frequency domain [47], [48]. This measure provides a bounded and normative evaluation of association, ranging between 0 and 1, with 0 in the case of linear independence, and 1 in the case of a perfect linear relationship.

Coherence between two signals is defined as the modulus of the *coherency*, a parameter that is estimated by computing the cross-power spectral density between the two random processes, divided by the square root of the product of their power spectral densities. This measure results in a complex value whose magnitude (coherence) and phase can be used to assess the existence of functional interactions. Since the activity of a single generator within the brain is typically observable in many points, with details of this mapping depending on the volume conductor [49], it is likely that a relation between channels is rather a trivial *volume conduction artefact* than a reflection of an underlying interacting brain [47]. For electrophysiological recordings one always needs a reference. If this reference is the same for the electrode pairs being studied, it can contribute significantly to the coherence, and thus, relative power changes may also affect coherencies without reflecting a change in *interaction* [50], [51]. Interestingly, it can be shown that the -complex- coherency of non-interacting sources is necessarily real and, hence, the imaginary part of coherency provides an excellent candidate to study -actual- interactions. The fundamental assumption to arrive at this conclusion is that the the quasi-static approximation holds, i.e. that an observed potential has no time-lag to the underlying source activity, which is indeed widely accepted [52]. The imaginary part of coherency is only sensitive to synchronizations of two processes which are time-lagged to each other. If volume conduction does not cause a time-lag, the imaginary part of coherency is hence insensitive to artifactual *self-interaction* [46].

As for the spectral variations, time-varying changes in the functional relationships between the different locations were assessed by computing -local- estimations of iCoh in sliding windows (blocks) of 60 seconds with 20 seconds of overlap.

For the imaginary coherence, the statistics were derived from that of the coherence. It is possible to define a stabilizing 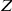-transform for the coherency 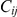, that becomes a scale transformation in the complex plane:
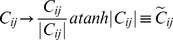
(4)where 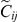 results to have in an approximately two dimensional Gaussian distribution in the complex plane. As a result, the variance of the imaginary part can be estimated according to:
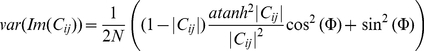
(5)where 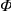 is the phase of 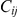, and 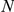 is the number of averaged windows used to estimate the power spectral densities.

### Statistical analysis

To assess the statistical significance of our results, mean power/iCoh along time of low gamma (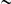
50 Hz), high gamma (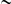
80 Hz) and HFO (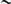
150 Hz) bands was calculated. The bandwidth of each band was chosen by selecting the mean frequency of the peaks in that band (in the four conditions: saline, and three doses of ketamine) and dividing it by a Q factor of 2.5 (BW = mean freq/Q). As a result, the average power/iCoh for the low gamma band was computed in the [40–60] Hz frequency range, in the [68–102] Hz range for the high gamma and between [128–192] Hz for the HFO band.

These mean values of power and iCoh were transformed as described by van Albada et al. [43] in order to ensure that they obey a Gaussian normal distribution.

Then, changes in -transformed- power and iCoh were assessed by means of repeated measures two-way ANOVAs where time (in steeps of 1 minute, from 15 minutes before injection until 60 minutes after) and dose (saline, 10, 25 and 50 mg/Kg) were taken as factors. Finally, Bonferroni post-hoc test was used to study significant differences between the three ketamine doses with respect to the saline condition along time.

Correlations between normalized power/iCoh values and locomotor activity were measured by means of the Pearson correlation coefficient.
